# Supplementary material for: Network approaches and interventions in healthcare settings: A systematic scoping review
Source: PLoS One. 2023 Feb 23;18(2):e0282050. doi: 10.1371/journal.pone.0282050 (PMC9949682; doi:10.1371/journal.pone.0282050)
Supplement: S1 Table — (PDF) [file pone.0282050.s001.pdf]

S1 Table. Database results

Databases	Current Search			Chambers et al (1)	
	Date searched	Date range searched	Records found	Date range searched	Records found
Embase	01.05.22	01.01.2010 – 01.05.2022	10,488	1980 – 2010 wk 52	1515
Medline	01.05.22	01.01.2010 – 01.05.2022	5,831	1948 – 30/12/10	1692
APA PsycINFO	01.05.22	01.01.2010 – 01.05.2022	5,098	1806 – Jan wk 1 2011	2292
Cochrane	01.05.22	01.01.2010 – 01.05.2022	366	4/10/10 and 22/12/10	48
Business Source Ultimate	01.05.22	01.01.2010 – 01.05.2022	6,170	Inception – 9/1/11	1214
Social Sciences Citation Index (SSCI) Conference Proceedings Citation Index – Social Science & Humanities (CPCI-SSH)	01.05.22	01.01.2010 – 01.05.2022	8,695	1956- present	883
CINAHL	01.05.22	01.01.2010 – 01.05.2022	3,442	1980 – 10/12/10	692
Additional papers found by reference checking and hand searching		Throughout	14		13
Health Management Information Consortium (HMIC)	01.05.22	01.01.2010 – 01.05.2022	198	Inception – Nov 2010	371
Social Network Journal	01.05.22	01.01.2010 – 01.05.2022	32		
