# Supplementary material for: Network approaches and interventions in healthcare settings: A systematic scoping review
Source: PLoS One. 2023 Feb 23;18(2):e0282050. doi: 10.1371/journal.pone.0282050 (PMC9949682; doi:10.1371/journal.pone.0282050)
Supplement: S2 Table — (PDF) [file pone.0282050.s002.pdf]

**S2 Table. Types of network ties**

| Types of ties | Words included | | | | | |
|---|---|---|---|---|---|---|
| Advice/mentoring | Advice | Advice seeking | Mentee-mentor relationships | | | |
| Information and knowledge exchange/sharing/transfer | Information exchange | Knowledge sharing | Information sharing | Knowledge exchange | knowledge transfer | Influence |
| Communication/discussion | communication | Discussion | Supportive supervision | Email communication | Patterns of team interaction | |
| Two-mode networks | Affiliation | Individual affiliation to surgical cases | Shared benefactors | 2-mode network of forum users by discussion threads | 2-mode networks of professionals by services provided | |
| Coordination/collaboration | Coordination | Cooperation | Collaboration | | | |
| Problem solving | Problem solving | Problem solving | | | | |
| Patient sharing/referrals | Patient-sharing physicians peer groups | Patient-related contacts | Referrals | | | |
| Physical contact/interaction | Physical contact or interaction | Face-to-face | | | | |
| Professional/work relationships | Professional interaction | Work related interactions | Partner Familiarity | Working relationships | Professional interactions | |
| Personal and social support | Social Support | Mutual support | Mutual understanding | Socialization | Friendship | Personal support |
