# Supplementary material for: Network approaches and interventions in healthcare settings: A systematic scoping review
Source: PLoS One. 2023 Feb 23;18(2):e0282050. doi: 10.1371/journal.pone.0282050 (PMC9949682; doi:10.1371/journal.pone.0282050)
Supplement: S3 Table — (PDF) [file pone.0282050.s003.pdf]

S3 Table. Network Measures

**Network Measures**	**Words included**
Density	Network density, inclusiveness.
Average path length	Distance, shortest path length.
Diameter	Network diameter.
Reciprocity/reciprocation	Reciprocity, in-degree /out-degree reciprocity.
Centrality Measure	Network centrality, degree centrality, closeness centrality, betweenness centrality, number of contacts, first degree centrality, second degree centrality, degree, degree betweenness, eigenvector centrality, betweenness, closeness, indegree centrality, eigenvector, outdegree centrality.
Network visualization	Visual sociograms, visual network graphs, network graph
Exponential Random Graph Models (ERGMs)	Activity/popularity, Assortativity.
Centralization	In-degree centralization, flow-betweenness centralization, network centralization.
Group centrality	Net degree of team, group betweenness, within-team density, within-team degree centrality, within-team cohesion, average number of ties.
MRQAP/Regression	Likelihood of information exchange, strength of collaboration tie.
Frequency/type of relation	frequency, type of communication, familiarity.
Core/periphery	Coreness, core/periphery structure.
E/I ratio	Numbers of internal/external ties, in-group/out-group interactions.
Geodesic distance	Average (geodesic) distance.
Contribution index	
Transitivity	
Connectivity	

Structural holes	
Group density	Sub-group cohesion, density by groups, in-group density.
Content analysis/SNA-based text mining	Content analysis.
Prestige	
Cliques	Clique identification.
Clustering	Cluster analysis (K-means), clustering, clustering coefficient.
Ego-network analysis (peer beliefs)	
Diffusion/contagion models	Regression, diffusion models.
SAOMs	Stochastic Actor-Oriented Models.
Tie strength reciprocity	
Group reciprocation	Within-team Reciprocation.
Hierarchy	Network authority
Fragmentation	
Isolates	
Brokerage roles	
Agent-based simulation	
Network size	
K-core/k-shell decomposition	K-shell centrality.
Components	
