# Supplementary material for: Network approaches and interventions in healthcare settings: A systematic scoping review
Source: PLoS One. 2023 Feb 23;18(2):e0282050. doi: 10.1371/journal.pone.0282050 (PMC9949682; doi:10.1371/journal.pone.0282050)
Supplement: S1 File — (PDF) [file pone.0282050.s004.pdf]

S1 File. Search Strategy. For 1st May 2022 and followed by January 2021

May 2022:

1. The Cochrane Library
   Wiley https://www.cochranelibrary.com

Cochrane Database of Systematic Reviews (CDSR), Issue 5 of 12, may 2022

Cochrane Protocols, Issue 5 of 12, January 2022 (one journal)

Cochrane Central Register of Controlled Trials (CENTRAL), Issue 4 of 12, April 2022

(62 journals)

Searched on: 01/05/2022
Search period: 2021 till current Cochrane
Records Identified: 64
Search Name: 2021 till current

#1 MeSH descriptor: [Interprofessional Relations] explode all trees 569
#2 (network or networks):ti,ab 16766
#3 (#1 and #2) 18
#4 ((social next network) near/3 (analy* or investigat* or examin* or explor* or inquir*)):ti,ab 81
#5 ((social next networks) near/3 (analy* or investigat* or examin* or explor* or inquir*)):ti,ab 22
#6 ((social next network) near/3 (theor* or framework* or model* or approach* or pattern* or perspective*)):ti,ab 36
#7 ((social next networks) near/3 (theor* or framework* or model* or approach* or pattern* or perspective*)):ti,ab 12
#8 ((social next network) near/3 (method* or technique* or tool* or survey* or questionnaire* or diagram* or software or data or dataset* or measure* or metric* or indicator* or information)):ti,ab 99
#9 ((social next networks) near/3 (method* or technique* or tool* or survey* or questionnaire* or diagram*or software or data or dataset* or measur* or metric* or indicator* or information)):ti,ab 46
#10 ((social next network) near/3 (intervention* or stud*)):ti,ab 178
#11 ((social next networks) near/3 (intervention* or stud*)):ti,ab 67
#12 MeSH descriptor: [Sociometric Techniques] this term only 30
#13 (sociometr* or sociogram* or sociomap*):ti,ab 53
#14 (opinion next leader*):ti,ab 250
#15 (#12 or #13 or #14) 322
#16 (#15 and #2) 44
#17 (UNICET or NetDraw or Pajek or KrackPlot):ti,ab 1
#18 (#3 or #4 or #5 or #6 or #7 or #8 or #9 or #10 #11 or #16 or #17) with **Publication Year from 2021 to present 64**

2. Ovid MEDLINE(R) ALL 1946 to April 29, 2022
OvidSP https://ovidsp.ovid.com
Search period: 2021 to present
Searched on: 1st May 2022

Records retrieved: 1109

1 exp Interprofessional Relations/ (71953)
2 (network or networks).ti,ab. (589286)
3 1 and 2 (1817)
4 ((social network or social networks) adj3 (analy$ or investigat$ or examin$ or explor$ or inquir$)).ti,ab. (3044)
5 ((social network or social networks) adj3 (theor$ or framework$ or model$ or approach$ or pattern$ or perspective$)).ti,ab. (1080)
6 ((social network or social networks) adj3 (method$ or technique$ or tool$ or survey$ or questionnaire$ or diagram$ or software or data or dataset$ or measur$ or metric$ or indicator$ or information)).ti,ab. (2098)
7 ((social network or social networks) adj3 (intervention$ or stud$)).ti,ab. (1124)
8 Sociometric Techniques/ (1181)
9 (sociometr$ or sociogram$ or sociomap$).ti,ab. (913)
10 opinion leader$.ti,ab. (1664)
11 8 or 9 or 10 (3427)
12 11 and 2 (405)
13 UCINET.ti,ab. (129)
14 NetDraw.ti,ab. (47)
15 Pajek.ti,ab. (47)

16 KrackPlot.ti,ab. (1)
17 3 or 4 or 5 or 6 or 7 or 12 or 13 or 14 or 15 or 16 (7599)
18 exp animals/ not humans.sh. (5001776)

19 17 not 18 (7296)
20 limit 19 to yr="2010 -Current" (**1109**)

3. Embase Classic+Embase 1947 to 2022 April 29

OvidSP https://ovidsp.ovid.com

Search period: 2021 to present

Searched on: 01.05.2022

Records identified: 1619

1 interdisciplinary communication/ (13133)

2 doctor nurse relation/ (7272)

3 1 or 2 (20257)

4 (network or networks).ti,ab. (719693)

5 3 and 4 (730)

6 *social network/ (6238)

7 ((social network or social networks) adj3 (analy$ or investigat$ or examin$ or explor$ or inquir$)).ti,ab. (3182)

8 ((social network or social networks) adj3 (theor$ or framework$ or model$ or approach$ or pattern$ or perspective$)).ti,ab. (1103)

9 ((social network or social networks) adj3 (method$ or technique$ or tool$ or survey$ or questionnaire$ or diagram$ or software or data or dataset$ or measur$ or metric$ or indicator$ or information)).ti,ab. (2514)

10 ((social network or social networks) adj3 (intervention$ or stud$)).ti,ab. (1264)

11 sociometric status/ (1076)

12 (sociometr$ or sociogram$ or sociomap$).ti,ab. (1077)

13 opinion leader$.ti,ab. (2302)

14 11 or 12 or 13 (4037)

15 4 and 14 (464)

16 UCINET.ti,ab. (164)

17 NetDraw.ti,ab. (58)

18 Pajek.ti,ab. (53

19 KrackPlot.ti,ab. (1)

20 5 or 6 or 7 or 8 or 9 or 10 or 15 or 16 or 17 or 18 or 19 (11514)

21 limit 20 to yr="2010 -Current" (1619)

4. APA PsycINFO 1806 to April week 29, 2022

OvidSP https://ovidsp.ovid.com

Search period: 2021 to current

Searched on: 01.05.2022

Records retrieved: **474**

1 ((social network or social networks) adj3 (analy$ or investigat$ or examin$ or explor$ or inquir$)).ti,ab. (3699)

2 ((social network or social networks) adj3 (theor$ or framework$ or model$ or approach$ or pattern$ or perspective$)).ti,ab. (1947)

3 ((social network or social networks) adj3 (method$ or technique$ or tool$ or survey$ or questionnaire$ or diagram$ or softwareor data or dataset$ or measur$ or metric$ or indicator$ or information)).ti,ab. (1878)

4 ((social network or social networks) adj3 (intervention$ or stud$)).ti,ab. (1576)

5 exp sociometry/ (1182)

6 (sociometr$ or sociogram$ or sociomap$).ti,ab. (4840)

7 opinion leader$.ti,ab. (823)

8 5 or 6 or 7 (5891)

9 (network or networks).ti,ab. (144966)

10 8 and 9 (554)

11 UCINET.ti,ab. (66)

12 NetDraw.ti,ab. (20)

13 Pajek.ti,ab. (18)

14 KrackPlot.ti,ab. (1)

15 1 or 2 or 3 or 4 or 10 or 11 or 12 or 13 or 14 (7297)

16 limit 15 to yr="2010-current" (474)

5. HMIC Health Management Information Consortium 1979 to march 2022

OvidSP https://ovidsp.ovid.com

Searched on: 1.05.22

Search period: 2021– current

Records retrieved: 3

1 exp interprofessional relations/ (2550)

2 PROFESSIONAL RELATIONS/ or NURSE PROFESSIONAL RELATIONS/ or MANAGER MEDICAL STAFF RELATIONS/ or STAFF RELATIONS/ or NURSE DOCTOT RELATIONS/ (252)

3 1 or 2 (2751)

4 (network or networks).ti,ab. (5682)

5 3 and 4 (159)

6 social networks/ (358)

7 network analysis/ (23)

8 ((social network or social networks) adj3 (analy$ or investigat$ or examin$ or explor$ or inquir$)).ti,ab. (83)

9 ((social network or social networks) adj3 (theor$ or framework$ or model$ or approach$ or pattern$ or perspective$)).ti,ab. (15)

10 ((social network or social networks) adj3 (method$ or technique$ or tool$ or survey$ or questionnaire$ or diagram$ or software or data or dataset$ or measur$ or metric$ or indicator$ or information)).ti,ab. (51)

11 ((social network or social networks) adj3 (intervention$ or stud$)).ti,ab. (21)

12 (sociometr$ or sociogram$ or sociomap$).ti,ab. (11)

13 opinion leader$.ti,ab. (98)

14 12 or 13 (109)

15 14 and 4 (8)

16 UCINET.ti,ab. (2)

17 NetDraw.ti,ab. (0)

18 Pajek.ti,ab. (0)

19 KrackPlot.ti,ab. (0)

20 5 or 6 or 7 or 8 or 9 or 10 or 11 or 15 or 16 or 17 or 18 or 19 (598)

21 limit 20 to yr="2021 -Current" (3)

6. CINAHL Plus with full text

EBSCO http;//search.ebscohost.com/

Searched on: 01.05.2022

Records retrieved: 506

S41 S5 or S6 or S7 or S8 or S9 or S10 or S11 or S12 or S13 or S14 or S15 or S16 or S17 or S18 or S19 or S20 or S21 or S22 or S23 or S24 or S25 or S26 or S27 or S28 or S29 or S30 or S31 or S36 or S37 or S38 or S39 or S40

Limiters - Published Date: 20210101-20220531- (506)

S40 TI KrackPlot or AB KrackPlot (0)

S39 TI Pajek or AB Pajek (16)

S38 TI NetDraw or AB NetDraw (21)

S37 TI UCINET or AB UCINET (65)

S36 S4 and S35 (188)

S35 S32 or S33 or S34 (1,176)

S34 TI "opinion leader*" or AB "opinion leader*" (797)

S33 TI (sociometr* or sociogram* or sociomap*) or AB (sociometr* or sociogram* or sociomap*) (284)

S32 (MH "Sociometric Techniques") (148)

S31 TI (“social network#” N3 stud*) or AB (“social network#” N3 stud*) (632)

S30 TI (“social network#” N3 intervention*) or AB (“social network#” N3 intervention*) (266)

S29 TI (“social network#” N3 information) or AB (“social network#” N3 information) (244)

S28 TI (“social network#” N3 indicator*) or AB (“social network#” N3 indicator*) (40)

S27 TI (“social network#” N3 metric*) or AB (“social network#” N3 metric*) (20)

S26 TI (“social network#” N3 measur*) or AB (“social network#” N3 measur*)

Search modes - Boolean/Phrase (248)

S25 TI (“social network#” N3 dataset*) or AB (“social network#” N3 dataset*) (3)

S24 TI (“social network#” N3 data) or AB (“social network#” N3 data) (341)

S23 TI (“social network#” N3 software) or AB (“social network#” N3 software) (19)

S22 TI (“social network#” N3 diagram*) or AB (“social network#” N3 diagram*) (20)

S21 TI (“social network#” N3 questionnaire*) or AB (“social network#” N3 questionnaire*) (65)

S20 TI (“social network#” N3 survey*) or AB (“social network#” N3 survey*) (120)

S19 TI (“social network#” N3 tool*) or AB (“social network#” N3 tool*) (80)

S18 TI (“social network#” N3 technique*) or AB (“social network#” N3 technique*) (41)

S17 TI (“social network#” N3 method*) or AB (“social network#” N3 method*) (327)

S16 TI (“social network#” N3 perspective*) or AB (“social network#” N3 perspective*) (87)

S15 TI (“social network#” N3 pattern*) or AB (“social network#” N3 pattern*) (82)

S14 TI (“social network#” N3 approach*) or AB (“social network#” N3 approach*) (175)

S13 TI (“social network#” N3 model*) or AB (“social network#” N3 model*) (159)

S12 TI (“social network#” N3 framework*) or AB (“social network#” N3 framework*) (43)

S11 TI (“social network#” N3 theor*) or AB (“social network#” N3 theor*)

(144)

S10 TI (“social network#” N3 inquir*) or AB (“social network#” N3 inquir*) (4)

S9 TI (“social network#” N3 explor*) or AB (“social network#” N3 explor*) (202)

S8 TI (“social network#” N3 examin*) or AB (“social network#” N3 examin*) (338)

S7 TI (“social network#” N3 investigat*) or AB (“social network#” N3 investigat*) (131)

S6 TI (“social network#” N3 analy*) or AB (“social network#” N3 analy*)

(1210)

S5 S3 and S4 (908)

S4 TI (network or networks) or AB (network or networks) (93,808)

S3 S1 or S2 (43,599)

S2 (MH "Intraprofessional Relations") (11,265)

S1 (MH "Interprofessional Relations+") (33,529)

7. Business Source Ultimate

EBSCO http;//search.ebscohost.com/

Searched on 01.05.2022

Records retrieved: 891

S40 S39 or S38 or S37 or S36 or S35 or S30 Limiters - Published Date: 20210101-20220531- (891)

S39 TI KrackPlot or AB KrackPlot (1)

S38 TI Pajek or AB Pajek (33)

S37 TI NetDraw or AB NetDraw (28)

S36 TI UCINET or AB UCINET (107)

S35 S34 and S2 (295)

S34 S33 or S32 or S31 (1,450)

S33 TI "opinion leader*" or AB "opinion leader*" (1026)

S32 DE "OPINION leaders" (46)

S31 TI (sociometr* or sociogram* or sociomap*) or AB (sociometr* or sociogram* or sociomap*) (419)

S30 S29 or S28 or S27 or S26 or S25 or S24 or S23 or S22 or S21 or S20 or S19 or S18 or S17 or S16 or S15 or S14 or S13 or S12 or S11 or S10 or S9 or S8 or S7 or S6 or S5 or S4 or S3 (7220)

S29 TI (“social network#” N3 stud*) or AB (“social network#” N3 stud*) (984)

S28 TI (“social network#” N3 intervention*) or AB (“social network#” N3 intervention*) (31)

S27 TI (“social network#” N3 information) or AB (“social network#” N3 information) (806)

S26 TI ("social network#" N3 indicator*) or AB ("social network#" N3 indicator*) (38)

S25 TI ("social network#" N3 metric*) or AB ("social network#" N3 metric*) (55)

S24 TI ("social network#" N3 measur*) or AB ("social network#" N3 measur*) (220)

S23 TI ("social network#" N3 dataset*) or AB ("social network#" N3 dataset*)

(61)

S22 TI ("social network#" N3 data) or AB ("social network#" N3 data) (746)

S21 TI ("social network#" N3 software) or AB ("social network#" N3 software) (103)

S20 TI ("social network#" N3 diagram*) or AB ("social network#" N3 diagram*) (4)

S19 TI ("social network#" N3 questionnaire*) or AB ("social network#" N3 questionnaire*) (27)

S18 TI ("social network#" N3 survey*) or AB ("social network#" N3 survey*) (182)

S17 TI ("social network#" N3 tool*) or AB ("social network#" N3 tool*) (266)

S16 TI ("social network#" N3 technique*) or AB ("social network#" N3 technique*) (177)

S15 TI ("social network#" N3 method*) or AB ("social network#" N3 method*) (486)

S14 TI ("social network#" N3 perspective*) or AB ("social network#" N3 perspective*) (437)

S13 TI ("social network#" N3 pattern*) or AB ("social network#" N3 pattern*) (144)

S12 TI ("social network#" N3 approach*) or AB ("social network#" N3 approach*) (388)

S11 TI ("social network#" N3 model*) or AB ("social network#" N3 model*) (540)

S10 TI (“social network#” N3 framework*) or AB (“social network#” N3 framework*) (130)

S9 TI (“social network#” N3 theor*) or AB (“social network#” N3 theor*) (806)

S8 TI (“social network#” N3 inquir*) or AB (“social network#” N3 inquir*) (2)

S7 TI (“social network#” N3 explor*) or AB (“social network#” N3 explor*) (278)

S6 TI (“social network#” N3 examin*) or AB (“social network#” N3 examin*) (412)

S5 TI (“social network#” N3 investigat*) or AB (“social network#” N3 investigat*) (232)

S4 TI (“social network#” N3 analy*) or AB (“social network#” N3 analy*) (2,738)

S3 S2 and S1 (229)

S2 TI (network or networks) or AB (network or networks) (621,853)

S1 DE "INTERPROFESSIONAL relations" (3,419)

8. Social Sciences Citation Index (SSCI)

Conference Proceedings Citation Index – Social Science & Humanities (CPCI-SSH)

Web of Science – ISI Web of Knowledge https://apps.webofknowledge.com/

SSCI 1970 – present

CPCI-SSH 1990 - present

Searched on 01.05.2022

Records retrieved: 411

66 411 #65 (restricted to 01-01-2021 to 01-05-2022)

65 12256 #64 OR #63 OR #62

64 10,790 #61 AND #11

63 2042 #60 AND #11

62 3955 #59 AND #11

61 1547038 #58 OR #57 OR #56 OR #55 OR #54 OR #53 OR #52 OR #51

60 283379 #50 OR #49 OR #48 OR #47 OR #46 OR #45 OR #44 OR #43 OR #42 OR #41 OR #40 OR #39 OR #38 OR #37 OR #36 OR #35 OR #34 OR #33 OR #32

59 647468 #31 OR #30 OR #29 OR #28 OR #27 OR #26 OR #25 OR #24 OR #23 OR #22 OR #21 OR #20 OR #19 OR #18 OR #17 OR #16 OR #15 OR #14 OR #13 OR #12

58 68456 TS=(pharmacy or pharmacies or hospice* or "health promotion" or "health education")

57 97302 TS=("primary care" or "primary health-care" or "primary healthcare" or PCT or "general practice*" or "GP practice*" or "GP surger*" or "group practice*" or "family practice*" or "family medicine" or "polyclinic*")

56 3420 TS=((acute or specialist or "National Health Service" or NHS) SAME trust*)

55 766799 TS=(hospital* or ward* or clinic* or outpatient* or "out-patient*" or "secondary care")

54 13011 TS=(dental SAME (setting* or service* or organi$ation* or system* or provider* or facility or facilities or institution* or trust* or center* or centre* or unit* or department* or practice* or surger* or sector* or industr* or group*))

53 447256 TS=((psychiatric or psycholog* or mental) SAME (setting* or service* or organi$ation* or system* or provider* or facility or facilities or institution* or trust* or center* or centre* or unit* or department* or practice* or sector* or industr* or group*))

52 454034 TS=((medical or clinical) SAME (setting* or service* or organi$ation* or system* or provider* or facility or facilities or institution* or trust* or center* or centre* or unit* or department* or practice* or sector* or industr* or group*))

51 816414 TS=((health or healthcare or health-care) SAME (setting* or service* or organi$ation* or system* or provider* or facility or facilities or institution* or trust* or center* or centre* or unit* or department* or practice* or sector* or industr* or group*))

50 3211 TS=(porter or porters or "ward attendant*" or "hospital auxiliar*" or "hospital orderl*")

49 2063 TS=((hospital* or health or medical or clinical) SAME (librarian* or "library assistant*" or "information officer*" or "information specialist*"))

48 35017 TS=((hospital* or health or medical or clinical) SAME (ceo or "chief executive*" or director or directors or leader*))

47 66033 TS=((administrator* or manager* or clerk* or clerical or secretar* or receptionist* or assistant*) SAME (health or healthcare or health-care or medical or hospital* or clinic* or ward* or outpatient* or out-patient* or practice*))

46 10686 TS=(office* SAME (personnel or staff or worker* or professional*or team* or assistant* or workforce*))

45 55876 TS=((managerial or management) SAME (personnel or staff or worker* or professional*or team* or officer* or assistant*or workforce*))

44 14,946 TS=((administrative or administration) SAME (personnel or staff or worker* or professional*or team* or officer* or assistant* or workforce*))

43 696 TS=(("art therapy" or "drama therapy" or "music therapy") SAME (personnel or staff or practitioner* or worker* or professional* or team* or officer* or assistant* or specialist* or workforce*))

42 777 TS=("art therapist*" or "drama therapist*" or "music therapist*")

41 57802 TS=((psychiatry or psychiatric or psychology or counselling or counseling or psychotherapy or psycho-therapy) SAME (personnel or staff or practitioner* or worker* or professional* or team* or officer* or assistant* or specialist* or workforce*))

40 60960 TS=(psychiatrist* or psychologist* or counsellor* or counselor* or psychotherapist* or psycho-therapist* or "psychiatric aide*" or "psychiatric assistant*")

39 355 TS=("clinical technician*" or "clinical technologist*" or "medical technician*" or "medical technologist*")

38 8360 TS=(("clinical engineering" or bioengineering or bio-engineering or biomedical or bio-medical or cardiography or cardiac or orthop$edic*) SAME (personnel or staff or practitioner* or worker* or professional* or team* or officer* or assistant* or specialist* or workforce*))

37 6 TS=(cardiographer* or "cardiological technician*" or "cardiological physiologist*" or "cardiac technician*" or "cardiac physiologist*")

36 962 TS= (bioengineer* or bio-engineer* or "biomedical engineer*" or "bio- medical engineer*" or "biomedical scientist*" or "bio-medical scientist*")

35 172 TS=("clinical cytogeneticist*" or "clinical embryologist*" or "clinical engineer*" or "clinical support worker*" or "clinical physiologist*" or "clinical scientist*")

34 9895 TS=(laboratory SAME (personnel or staff or practitioner* or worker* or professional* or team* or officer* or assistant* or specialist* or workforce* or scientist* or technician*))

33 8267 TS=((phlebotomy or microbiolog* or virolog* or cytolog* or biochemistry or bio-chemistry or gastrointestinal or gastroenterolog* or "clinical photography" or "medical photography" or radiograph* or sonograph* or radiotherapy or "medical physics" or "molecular genetics" or pharmacolog* or toxicology* or "respiratory physiology*" or "operating room" or "operating department") SAME (personnel or staff or practitioner* or worker* or professional* or team* or officer* or assistant* or specialist* or workforce*))

32 3159 TS=(phlebotomist* or microbiologist* or virologist* or "cytology screener*" or cytoscreener* or bio-chemist or bio-chemists or biochemist or biochemists or "gastrointestinal physiologist*" or "gastroenterology technician*" or "medical illustrator*" or "clinical photographer*" or "medical photographer*" or radiographer* or sonographer* or radiotherapist* or "medical physicist*" or perfusionist* or "molecular geneticist*" or pharmacologist* or toxicologist* or anatomist* or coroner* or "medical examiner*" or "respiratory physiologist*" or "respiratory physiology technician*" or "operating department technician*" or "operating room technician*" or "plaster technician*" or "physiological scientist*" or "clinical physiologist*" or "medical technical officer*")

31 418 TS=((podiatry or chiropody or orthotic* or prosthetic*) SAME (personnel or staff or practitioner* or worker* or professional* or team* or officer* or assistant* or specialist* or workforce*))

30 283 TS=(podiatrist* or chiropodist* or orthotist* or prosthetist*)

29 418 TS=(audiolog* SAME (personnel or staff or practitioner* or worker* or professional* or team* or officer* or assistant* or specialist* or workforce*))

28 466 TS=(audiologist* or "hearing therapist*" or "hearing specialist*" or "audiological scientist*")

27 138 TS=((optometry or orthoptic*or ophthalmic*) SAME (personnel or staff or practitioner* or worker* or professional* or team* or officer* or assistant* or specialist* or workforce*))

26 338 TS=(optician* or optometrist* or orthoptist* or "ophthalmic technician*" or "ophthalmic technologist*")

25 4734 TS=(pharmacy SAME (personnel or staff or practitioner* or worker* or professional* or team* or officer* or assistant* or specialist* or workforce*))

24 9494 TS=(pharmacist* or "pharmacy technician*" or "dispensing assistant*" or "dispensing technician*")

23 7407 TS=(dentist* or orthodontist*)

22 4804 TS=(dental SAME (personnel or staff or practitioner* or worker* or professional* or team* or officer* or assistant* or specialist* or workforce* or hygienist* or technician* or therapist* or auxiliar* or receptionist* or facult*))

21 4048 TS=(paramedic* or para-medic* or ambulance* or "patient transport")

20 35493 TS=((physiotherapy or "manual therapy" or "physical therapy" or "occupational therapy" or "speech therapy" or "speech and language therapy") SAME (personnel or staff or practitioner* or worker* or professional* or team* or officer* or assistant* or specialist* or workforce*))

19 9655 TS=(physiotherapist* or "manual therapist*" or "physical therapist*" or "occupational therapist*" or "speech therapist*" or "speech and language therapist*")

18 6316 TS=("allied health" or paraprofessional* or para-professional*)

17 15,328 TS=(midwife* or midwives or "birth attendant*" or "health visitor*" or "community practitioner*")

16 208941 TS= (nurse* or nursing)

15 40621 TS= ((an$esthetic or an$esthesia or cardiolog* or dermatolog* or endocrinolog* or gastroenterolog* or gyn$ecolog* or h$ematolog* or immunolog* or laryngolog* or obstetric* or ophthalmolog* or oncolog* or otolaryngolog* or otolog* or neonatolog* or neo-natolog* or neurolog* or neurosurgical or neuro-surgical or pathology* or p$ediatric* or nephrolog* or radiolog* or radiotherapy or rheumatolog* or rhinolog* or surgical or urolog* or neurophysiolog* or neuro-physiolog*) SAME (personnel or staff or practitioner* or worker* or professional* or team* or officer* or assistant* or specialist* or workforce*))

14 34224 TS=(an$esthetist* or cardiologist* or dermatologist* or endocrinologist* or gastroenterologist* or geriatrician* or gyn$ecologist* or h$ematologist* or hospitalist* or immunologist* or laryngologist* or obstetrician* or ophthalmologist* or oncologist* or otolaryngologist* or otologist* or neonatologist* or neo-natologist* or neurologist* or neurosurgeon* or neuro-surgeon* or pathologist* or p$ediatrician* or nephrologist* or

radiologist* or rheumatologist* or rhinologist* or surgeon* or urologist* or neurophysiologist* or neuro-physiologist*)

13 248796 TS=(doctor or doctors or physician* or clinician* or consultant* or "general practitioner*" or GP or generalist* or registrar* or "medical facult*" or "medical student*" or fellow* or "house officer*" or "family practitioner*")

12 339040 TS=((health or healthcare or health-care or medical or hospital* or ward* or clinic* or outpatient* or out-patient* or non-medical or non-clinical or "patient care") SAME (personnel or staff or practitioner* or worker* or professional* or team* or officer* or assistant* or specialist* or workforce*))

11 46214 #10 OR #9 OR #4 OR #3 OR #2 OR #1

10 332 TS=(UCINET or NetDraw or Pajek or KrackPlot)

9 1101 #8 AND #7

8 316955 TS=(network or networks)

7 4122 #6 OR #5

6 1,822 TS="opinion leader*"

5 2328 TS=(sociometr* or sociogram* or sociomap*)

4 31,831 TS=(("social network" or "social networks") SAME (intervention* or stud*))

3 23727 TS=(("social network" or "social networks") SAME (method* or technique* or tool* or survey* or questionnaire* or diagram* or software or data or dataset* or measur* or metric* or indicator* or information))

2 30817 TS=(("social network" or "social networks") SAME (theor* or framework* or model* or approach* or pattern* or perspective*))

1 36530 TS=(("social network" or "social networks") SAME (analy* or investigat* or examin* or explor* or inquir*))

Key

TS= topic tag; searches terms in title, abstract, author keywords and keywords plus fields

* = truncation

$ = wildcard, unknown character or no character

“ “ = phrase search

SAME = terms within same sentence

Below searches were ran up to January 2021.

1. The Cochrane Library **(2021:302 results)**
   Wiley https://www.cochranelibrary.com

Cochrane Database of Systematic Reviews (CDSR), Issue 1 of 12, January 2021

Cochrane Protocols, Issue 1 of 12, January 2021 (none)

Cochrane Central Register of Controlled Trials (CENTRAL), Issue 1 of 12, January 2021 (301 journals)

Searched on: **04/01/2021**
Search period: 2010 till current Cochrane
Records Identified: **302**
Search Name: 2010 till current

#1	MeSH descriptor: [Interprofessional Relations] explode all trees	569
#2	(network or networks):ti,ab	14020
#3	(#1 and #2)	16

#4 ((social next network) near/3 (analy* or investigat* or examin* or explor* or inquir*)):ti,ab 65
#5 ((social next networks) near/3 (analy* or investigat* or examin* or explor* or inquir*)):ti,ab 19
#6 ((social next network) near/3 (theor* or framework* or model* or approach* or pattern* or perspective*)):ti,ab 27
#7 ((social next networks) near/3 (theor* or framework* or model* or approach* or pattern* or perspective*)):ti,ab 10
#8 ((social next network) near/3 (method* or technique* or tool* or survey* or questionnaire* or diagram* or software or data or dataset* or measure* or metric* or indicator* or information)):ti,ab 79
#9 ((social next networks) near/3 (method* or technique* or tool* or survey* or questionnaire* or diagram*or software or data or dataset* or measur* or metric* or indicator* or information)):ti,ab 38
#10 ((social next network) near/3 (intervention* or stud*)):ti,ab 148
#11 ((social next networks) near/3 (intervention* or stud*)):ti,ab 57
#12 MeSH descriptor: [Sociometric Techniques] this term only 29
#13 (sociometr* or sociogram* or sociomap*):ti,ab 51
#14 (opinion next leader*):ti,ab 233
#15 (#12 or #13 or #14) 303
#16 (#15 and #2) 36
#17 (UNICET or NetDraw or Pajek or KrackPlot):ti,ab 1
#18 (#3 or #4 or #5 or #6 or #7 or #8 or #9 or #10 #11 or #16 or #17) with **Publication Year from 2010 to present 302**

2. Ovid MEDLINE(R) ALL 1946 to December 31, 2020
OvidSP https://ovidsp.ovid.com
Search period: 2010 to present
Searched on: 4th January 2021

Records retrieved: **4722**

1 exp Interprofessional Relations/ (70027)
2 (network or networks).ti,ab. (500630)
3 1 and 2 (1751)
4 ((social network or social networks) adj3 (analy$ or investigat$ or examin$ or explor$ or inquir$)).ti,ab. (2490)
5 ((social network or social networks) adj3 (theor$ or framework$ or model$ or approach$ or pattern$ or perspective$)).ti,ab. (906)
6 ((social network or social networks) adj3 (method$ or technique$ or tool$ or survey$ or questionnaire$ or diagram$ or software or data or dataset$ or measur$ or metric$ or indicator$ or information)).ti,ab. (1739)
7 ((social network or social networks) adj3 (intervention$ or stud$)).ti,ab. (929)
8 Sociometric Techniques/ (1176)
9 (sociometr$ or sociogram$ or sociomap$).ti,ab. (862)

10 opinion leader$.ti,ab. (1499)
11 8 or 9 or 10 (3210)
12 11 and 2 (353)
13 UCINET.ti,ab. (107)
14 NetDraw.ti,ab. (39)
15 Pajek.ti,ab. (40)

16 KrackPlot.ti,ab. (1)
17 3 or 4 or 5 or 6 or 7 or 12 or 13 or 14 or 15 or 16 (6537)
18 exp animals/ not humans.sh. (4771716)

19 17 not 18 (6303)
20 limit 19 to yr="2010 -Current" (**4722**)

3. Embase Classic+Embase 1947 to 2020 December 31

OvidSP https://ovidsp.ovid.com

Search period: 2010 to present

Searched on: 4th January 2021

Records identified: **8869**

1 interdisciplinary communication/ (12520)

2 doctor nurse relation/ (6198)

3 1 or 2 (18573)

4 (network or networks).ti,ab. (620001)

5 3 and 4 (656)

6 *social network/ (5820)

7 ((social network or social networks) adj3 (analy$ or investigat$ or examin$ or explor$ or inquir$)).ti,ab. (2673)

8 ((social network or social networks) adj3 (theor$ or framework$ or model$ or approach$ or pattern$ or perspective$)).ti,ab. (951)

9 ((social network or social networks) adj3 (method$ or technique$ or tool$ or survey$ or questionnaire$ or diagram$ or software or data or dataset$ or measur$ or metric$ or indicator$ or information)).ti,ab. (2143)

10 ((social network or social networks) adj3 (intervention$ or stud$)).ti,ab. (1086)

11 sociometric status/ (1056)

12 (sociometr$ or sociogram$ or sociomap$).ti,ab. (1033)

13 opinion leader$.ti,ab. (2088)

14 11 or 12 or 13 (3778)

15 4 and 14 (401)

16 UCINET.ti,ab. (141)

17 NetDraw.ti,ab. (51)

18 Pajek.ti,ab. (46)

19 KrackPlot.ti,ab. (1)

20 5 or 6 or 7 or 8 or 9 or 10 or 15 or 16 or 17 or 18 or 19 (10156)

21 limit 20 to yr="2010 -Current" (8869)

4. APA PsycINFO 1806 to December week 4 2020

OvidSP https://ovidsp.ovid.com

Search period: 2010 to current

Searched on: 4th January 2021

Records retrieved: **4624**

1 ((social network or social networks) adj3 (analy$ or investigat$ or examin$ or explor$ or inquir$)).ti,ab. (3335)

2 ((social network or social networks) adj3 (theor$ or framework$ or model$ or approach$ or pattern$ or perspective$)).ti,ab. (1784)

3 ((social network or social networks) adj3 (method$ or technique$ or tool$ or survey$ or questionnaire$ or diagram$ or softwareor data or dataset$ or measur$ or metric$ or indicator$ or information)).ti,ab. (1703)

4 ((social network or social networks) adj3 (intervention$ or stud$)).ti,ab. (1424)

5 exp sociometry/ (1146)

6 (sociometr$ or sociogram$ or sociomap$).ti,ab. (4758)

7 opinion leader$.ti,ab. (768)

8 5 or 6 or 7 (5749)

9 (network or networks).ti,ab. (131492)

10 8 and 9 (561)

11 UCINET.ti,ab. (64)

12 NetDraw.ti,ab. (20)

13 Pajek.ti,ab. (18)

14 KrackPlot.ti,ab. (1)

15 1 or 2 or 3 or 4 or 10 or 11 or 12 or 13 or 14 (6652)

16 limit 15 to yr="2010-current" (4624)

5. HMIC Health Management Information Consortium 1979 to November 2020

OvidSP https://ovidsp.ovid.com

Searched on: 4th January 2021

Search period: 2010 – current

Records retrieved: **195**

1 exp interprofessional relations/ (2526)

2 PROFESSIONAL RELATIONS/ or NURSE PROFESSIONAL RELATIONS/ or MANAGER MEDICAL STAFF RELATIONS/ or STAFF RELATIONS/ or NURSE DOCTOT RELATIONS/ (250)

3 1 or 2 (2725)

4 (network or networks).ti,ab. (5571)

5 3 and 4 (157)

6 social networks/ (356)

7 network analysis/ (23)

8 ((social network or social networks) adj3 (analy$ or investigat$ or examin$ or explor$ or inquir$)).ti,ab. (80)

9 ((social network or social networks) adj3 (theor$ or framework$ or model$ or approach$ or pattern$ or perspective$)).ti,ab. (15)

10 ((social network or social networks) adj3 (method$ or technique$ or tool$ or survey$ or questionnaire$ or diagram$ or software or data or dataset$ or measur$ or metric$ or indicator$ or information)).ti,ab. (50)

11 ((social network or social networks) adj3 (intervention$ or stud$)).ti,ab. (21)

12 (sociometr$ or sociogram$ or sociomap$).ti,ab. (11)

13 opinion leader$.ti,ab. (98)

14 12 or 13 (109)

15 14 and 4 (8)

16 UCINET.ti,ab. (2)

17 NetDraw.ti,ab. (0)

18 Pajek.ti,ab. (0)

19 KrackPlot.ti,ab. (0)

20 5 or 6 or 7 or 8 or 9 or 10 or 11 or 15 or 16 or 17 or 18 or 19 (592)

21 limit 20 to yr="2010 -Current" (4)

As the above didn't reflect previous searches, I didn't limit the date and instead ran (20) for all publication dates and instead I went through the 592 paper individually and there were 195 within 2010 to current.

6. CINAHL

EBSCO http;//search.ebscohost.com/

Searched on 4th January 2021

Records retrieved: **2936**

S41 S5 or S6 or S7 or S8 or S9 or S10 or S11 or S12 or S13 or S14 or S15 or S16 or S17 or S18 or S19 or S20 or S21 or S22 or S23 or S24 or S25 or S26 or S27 or S28 or S29 or S30 or S31 or S36 or S37 or S38 or S39 or S40

Limiters - Published Date: 20100101- (2,936)

S40 TI KrackPlot or AB KrackPlot (0)

S39 TI Pajek or AB Pajek (14)

S38 TI NetDraw or AB NetDraw (15)

S37 TI UCINET or AB UCINET (49)

S36 S4 and S35 (153)

S35 S32 or S33 or S34 (1,059)

S34 TI "opinion leader*" or AB "opinion leader*" (712)

S33 TI (sociometr* or sociogram* or sociomap*) or AB (sociometr* or sociogram* or sociomap*) (253)

S32 (MH "Sociometric Techniques") (141)

S31 TI ("social network#" N3 stud*) or AB ("social network#" N3 stud*) (547)

S30 TI ("social network#" N3 intervention*) or AB ("social network#" N3 intervention*) (218)

S29 TI ("social network#" N3 information) or AB ("social network#" N3 information) (209)

S28 TI ("social network#" N3 indicator*) or AB ("social network#" N3 indicator*) (35)

S27 TI ("social network#" N3 metric*) or AB ("social network#" N3 metric*) (18)

S26 TI ("social network#" N3 measur*) or AB ("social network#" N3 measur*)

Search modes - Boolean/Phrase (219)

S25 TI ("social network#" N3 dataset*) or AB ("social network#" N3 dataset*) (2)

S24 TI ("social network#" N3 data) or AB ("social network#" N3 data) (289)

S23 TI ("social network#" N3 software) or AB ("social network#" N3 software) (16)

S22 TI (“social network#” N3 diagram*) or AB (“social network#” N3 diagram*) (17)

S21 TI (“social network#” N3 questionnaire*) or AB (“social network#” N3 questionnaire*) (61)

S20 TI (“social network#” N3 survey*) or AB (“social network#” N3 survey*) (100)

S19 TI (“social network#” N3 tool*) or AB (“social network#” N3 tool*) (65)

S18 TI (“social network#” N3 technique*) or AB (“social network#” N3 technique*) (36)

S17 TI (“social network#” N3 method*) or AB (“social network#” N3 method*) (287)

S16 TI (“social network#” N3 perspective*) or AB (“social network#” N3 perspective*) (78)

S15 TI (“social network#” N3 pattern*) or AB (“social network#” N3 pattern*) (75)

S14 TI (“social network#” N3 approach*) or AB (“social network#” N3 approach*) (146)

S13 TI (“social network#” N3 model*) or AB (“social network#” N3 model*) (136)

S12 TI (“social network#” N3 framework*) or AB (“social network#” N3 framework*) (34)

S11 TI (“social network#” N3 theor*) or AB (“social network#” N3 theor*)

(128)

S10 TI (“social network#” N3 inquir*) or AB (“social network#” N3 inquir*) (3)

S9 TI (“social network#” N3 explor*) or AB (“social network#” N3 explor*) (172)

S8 TI (“social network#” N3 examin*) or AB (“social network#” N3 examin*) (300)

S7 TI (“social network#” N3 investigat*) or AB (“social network#” N3 investigat*) (113)

S6 TI (“social network#” N3 analy*) or AB (“social network#” N3 analy*)

(1,029)

S5 S3 and S4 (841)

S4 TI (network or networks) or AB (network or networks) (78,925)

S3 S1 or S2 (40,549)

S2 (MH "Intraprofessional Relations") (10,835)

S1 (MH "Interprofessional Relations+") (30,850)

7. Business Source Ultimate

EBSCO http;//search.ebscohost.com/

Searched on 5th January 2021

Records retrieved: **5279**

S40 S39 or S38 or S37 or S36 or S35 or S30 Limiters - Published Date: 20100101- (5,279)

S39 TI KrackPlot or AB KrackPlot (1)

S38 TI Pajek or AB Pajek (29)

S37 TI NetDraw or AB NetDraw (20)

S36 TI UCINET or AB UCINET (86)

S35 S34 and S2 (274)

S34 S33 or S32 or S31 (1,348)

S33 TI "opinion leader*" or AB "opinion leader*" (942)

S32 DE "OPINION leaders" (40)

S31 TI (sociometr* or sociogram* or sociomap*) or AB (sociometr* or sociogram* or sociomap*) (403)

S30 S29 or S28 or S27 or S26 or S25 or S24 or S23 or S22 or S21 or S20 or S19 or S18 or S17 or S16 or S15 or S14 or S13 or S12 or S11 or S10 or S9 or S8 or S7 or S6 or S5 or S4 or S3 (6,318)

S29 TI (“social network#” N3 stud*) or AB (“social network#” N3 stud*) (855)

S28 TI (“social network#” N3 intervention*) or AB (“social network#” N3 intervention*) (25)

S27 TI (“social network#” N3 information) or AB (“social network#” N3 information) (729)

S26 TI (“social network#” N3 indicator*) or AB (“social network#” N3 indicator*) (32)

S25 TI (“social network#” N3 metric*) or AB (“social network#” N3 metric*) (50)

S24 TI (“social network#” N3 measur*) or AB (“social network#” N3 measur*) (191)

S23 TI (“social network#” N3 dataset*) or AB (“social network#” N3 dataset*)

(49)

S22 TI (“social network#” N3 data) or AB (“social network#” N3 data) (647)

S21 TI (“social network#” N3 software) or AB (“social network#” N3 software) (101)

S20 TI (“social network#” N3 diagram*) or AB (“social network#” N3 diagram*) (4)

S19 TI (“social network#” N3 questionnaire*) or AB (“social network#” N3 questionnaire*) (22)

S18 TI (“social network#” N3 survey*) or AB (“social network#” N3 survey*) (162)

S17 TI (“social network#” N3 tool*) or AB (“social network#” N3 tool*) (241)

S16 TI (“social network#” N3 technique*) or AB (“social network#” N3 technique*) (154)

S15 TI (“social network#” N3 method*) or AB (“social network#” N3 method*) (409)

S14 TI (“social network#” N3 perspective*) or AB (“social network#” N3 perspective*) (394)

S13 TI (“social network#” N3 pattern*) or AB (“social network#” N3 pattern*) (102)

S12 TI (“social network#” N3 approach*) or AB (“social network#” N3 approach*) (314)

S11 TI (“social network#” N3 model*) or AB (“social network#” N3 model*) (443)

S10 TI (“social network#” N3 framework*) or AB (“social network#” N3 framework*) (117)

S9 TI (“social network#” N3 theor*) or AB (“social network#” N3 theor*) (724)

S8 TI (“social network#” N3 inquir*) or AB (“social network#” N3 inquir*) (2)

S7 TI (“social network#” N3 explor*) or AB (“social network#” N3 explor*) (250)

S6 TI (“social network#” N3 examin*) or AB (“social network#” N3 examin*) (380)

S5 TI (“social network#” N3 investigat*) or AB (“social network#” N3 investigat*) (203)

S4 TI (“social network#” N3 analy*) or AB (“social network#” N3 analy*) (2,310)

S3 S2 and S1 (208)

S2 TI (network or networks) or AB (network or networks) (582,940)

S1 DE "INTERPROFESSIONAL relations" (3,062)

8. Social Sciences Citation Index (SSCI)

Conference Proceedings Citation Index – Social Science & Humanities (CPCI-SSH)

Web of Science – ISI Web of Knowledge https://apps.webofknowledge.com/

SSCI 1970 – present

CPCI-SSH 1990 - present

Searched on 28th December 2020

Records retrieved: **8284**

66 8,284 #65

65 8,284 #64 OR #63 OR #62

64 7,250 #61 AND #11

63 1,458 #60 AND #11

62 2,702 #59 AND #11

61 870,432 #58 OR #57 OR #56 OR #55 OR #54 OR #53 OR #52 OR #51

60 159,187 #50 OR #49 OR #48 OR #47 OR #46 OR #45 OR #44 OR #43 OR #42 OR #41 OR #40 OR #39 OR #38 OR #37 OR #36 OR #35 OR #34 OR #33 OR #32

59 376,495 #31 OR #30 OR #29 OR #28 OR #27 OR #26 OR #25 OR #24 OR #23 OR #22 OR #21 OR #20 OR #19 OR #18 OR #17 OR #16 OR #15 OR #14 OR #13 OR #12

58 39,262 TS=(pharmacy or pharmacies or hospice* or "health promotion" or "health education")

57 56,505 TS=("primary care" or "primary health-care" or "primary healthcare" or PCT or "general practice*" or "GP practice*" or "GP surger*" or "group practice*" or "family practice*" or "family medicine" or "polyclinic*")

56 2,250 TS=((acute or specialist or "National Health Service" or NHS) SAME trust*)

55 421,482 TS=(hospital* or ward* or clinic* or outpatient* or "out-patient*" or "secondary care")

54 7,851 TS=(dental SAME (setting* or service* or organi$ation* or system* or provider* or facility or facilities or institution* or trust* or center* or centre* or unit* or department* or practice* or surger* or sector* or industr* or group*))

53 250,220 TS=((psychiatric or psycholog* or mental) SAME (setting* or service* or organi$ation* or system* or provider* or facility or facilities or institution* or trust* or center* or centre* or unit* or department* or practice* or sector* or industr* or group*))

52 265,104 TS=((medical or clinical) SAME (setting* or service* or organi$ation* or system* or provider* or facility or facilities or institution* or trust* or center* or centre* or unit* or department* or practice* or sector* or industr* or group*))

51 499,042 TS=((health or healthcare or health-care) SAME (setting* or service* or organi$ation* or system* or provider* or facility or facilities or institution* or trust* or center* or centre* or unit* or department* or practice* or sector* or industr* or group*))

50 1,129 TS=(porter or porters or "ward attendant*" or "hospital auxiliar*" or "hospital orderl*")

49 985 TS=((hospital* or health or medical or clinical) SAME (librarian* or "library assistant*" or "information officer*" or "information specialist*"))

48 22,302 TS=((hospital* or health or medical or clinical) SAME (ceo or "chief executive*" or director or directors or leader*))

47 40,495 TS=((administrator* or manager* or clerk* or clerical or secretar* or receptionist* or assistant*) SAME (health or healthcare or health-care or medical or hospital* or clinic* or ward* or outpatient* or out-patient* or practice*))

46 6,112 TS=(office* SAME (personnel or staff or worker* or professional*or team* or assistant* or workforce*))

45 34,003 TS=((managerial or management) SAME (personnel or staff or worker* or professional*or team* or officer* or assistant*or workforce*))

44 8,802 TS=((administrative or administration) SAME (personnel or staff or worker* or professional*or team* or officer* or assistant* or workforce*))

43 444 TS=(("art therapy" or "drama therapy" or "music therapy") SAME (personnel or staff or practitioner* or worker* or professional* or team* or officer* or assistant* or specialist* or workforce*))

42 418 TS=("art therapist*" or "drama therapist*" or "music therapist*")

41 30,430 TS=((psychiatry or psychiatric or psychology or counselling or counseling or psychotherapy or psycho-therapy) SAME (personnel or staff or practitioner* or worker* or professional* or team* or officer* or assistant* or specialist* or workforce*))

40 26,097 TS=(psychiatrist* or psychologist* or counsellor* or counselor* or psychotherapist* or psycho-therapist* or "psychiatric aide*" or "psychiatric assistant*")

39 180 TS=("clinical technician*" or "clinical technologist*" or "medical technician*" or "medical technologist*")

38 5,341 TS=(("clinical engineering" or bioengineering or bio-engineering or biomedical or bio-medical or cardiography or cardiac or orthop$edic*) SAME (personnel or staff or practitioner* or worker* or professional* or team* or officer* or assistant* or specialist* or workforce*))

37 6 TS=(cardiographer* or "cardiological technician*" or "cardiological physiologist*" or "cardiac technician*" or "cardiac physiologist*")

36 606 TS= (bioengineer* or bio-engineer* or "biomedical engineer*" or "bio- medical engineer*" or "biomedical scientist*" or "bio-medical scientist*")

35 76 TS=("clinical cytogeneticist*" or "clinical embryologist*" or "clinical engineer*" or "clinical support worker*" or "clinical physiologist*" or "clinical scientist*")

34 6,011 TS=(laboratory SAME (personnel or staff or practitioner* or worker* or professional* or team* or officer* or assistant* or specialist* or workforce* or scientist* or technician*))

33 5,138 TS=((phlebotomy or microbiolog* or virolog* or cytolog* or biochemistry or bio-chemistry or gastrointestinal or gastroenterolog* or "clinical photography" or "medical photography" or radiograph* or sonograph* or radiotherapy or "medical physics" or "molecular genetics" or pharmacolog* or toxicology* or "respiratory physiology*" or "operating room" or "operating department") SAME (personnel or staff or practitioner* or worker* or professional* or team* or officer* or assistant* or specialist* or workforce*))

32 1,625 TS=(phlebotomist* or microbiologist* or virologist* or "cytology screener*" or cytoscreener* or bio-chemist or bio-chemists or biochemist or biochemists or "gastrointestinal physiologist*" or "gastroenterology technician*" or "medical illustrator*" or "clinical photographer*" or "medical photographer*" or radiographer* or sonographer* or radiotherapist* or "medical physicist*" or perfusionist* or "molecular geneticist*" or pharmacologist* or toxicologist* or anatomist* or coroner* or "medical examiner*" or "respiratory physiologist*" or "respiratory physiology technician*" or "operating department technician*" or "operating room technician*" or "plaster technician*" or "physiological scientist*" or "clinical physiologist*" or "medical technical officer*")

31 280 TS=((podiatry or chiropody or orthotic* or prosthetic*) SAME (personnel or staff or practitioner* or worker* or professional* or team* or officer* or assistant* or specialist* or workforce*))

30 176 TS=(podiatrist* or chiropodist* or orthotist* or prosthetist*)

29 276 TS=(audiolog* SAME (personnel or staff or practitioner* or worker* or professional* or team* or officer* or assistant* or specialist* or workforce*))

28 303 TS=(audiologist* or "hearing therapist*" or "hearing specialist*" or "audiological scientist*")

27 85 TS=((optometry or orthoptic*or ophthalmic*) SAME (personnel or staff or practitioner* or worker* or professional* or team* or officer* or assistant* or specialist* or workforce*))

26 179 TS=(optician* or optometrist* or orthoptist* or "ophthalmic technician*" or "ophthalmic technologist*")

25 3,120 TS=(pharmacy SAME (personnel or staff or practitioner* or worker* or professional* or team* or officer* or assistant* or specialist* or workforce*))

24 6,011 TS=(pharmacist* or "pharmacy technician*" or "dispensing assistant*" or "dispensing technician*")

23 4,152 TS=(dentist* or orthodontist*)

22 3,001 TS=(dental SAME (personnel or staff or practitioner* or worker* or professional* or team* or officer* or assistant* or specialist* or workforce* or hygienist* or technician* or therapist* or auxiliar* or receptionist* or facult*))

21 2,467 TS=(paramedic* or para-medic* or ambulance* or "patient transport")

20 3,509 TS=((physiotherapy or "manual therapy" or "physical therapy" or "occupational therapy" or "speech therapy" or "speech and language therapy") SAME (personnel or staff or practitioner* or worker* or professional* or team* or officer* or assistant* or specialist* or workforce*))

19 6,038 TS=(physiotherapist* or "manual therapist*" or "physical therapist*" or "occupational therapist*" or "speech therapist*" or "speech and language therapist*")

18 3,716 TS=("allied health" or paraprofessional* or para-professional*)

17 8,971 TS=(midwife* or midwives or "birth attendant*" or "health visitor*" or "community practitioner*")

16 115,000 TS= (nurse* or nursing)

15 25,719 TS= ((an$esthetic or an$esthesia or cardiolog* or dermatolog* or endocrinolog* or gastroenterolog* or gyn$ecolog* or h$ematolog* or immunolog* or laryngolog* or obstetric* or ophthalmolog* or oncolog* or otolaryngolog* or otolog* or neonatolog* or neo-natolog* or neurolog* or neurosurgical or neuro-surgical or pathology* or p$ediatric* or nephrolog* or radiolog* or radiotherapy or rheumatolog* or rhinolog* or surgical or urolog* or neurophysiolog* or neuro-physiolog*) SAME (personnel or staff or practitioner* or worker* or professional* or team* or officer* or assistant* or specialist* or workforce*))

14 19,710 TS=(an$esthetist* or cardiologist* or dermatologist* or endocrinologist* or gastroenterologist* or geriatrician* or gyn$ecologist* or h$ematologist* or hospitalist* or immunologist* or laryngologist* or obstetrician* or ophthalmologist* or oncologist* or otolaryngologist* or otologist* or neonatologist* or neo-natologist* or neurologist* or neurosurgeon* or neuro-surgeon* or pathologist* or p$ediatrician* or nephrologist* or

radiologist* or rheumatologist* or rhinologist* or surgeon* or urologist* or neurophysiologist* or neuro-physiologist*)

13 132,859 TS=(doctor or doctors or physician* or clinician* or consultant* or "general practitioner*" or GP or generalist* or registrar* or "medical facult*" or "medical student*" or fellow* or "house officer*" or "family practitioner*")

12 203,464 TS=((health or healthcare or health-care or medical or hospital* or ward* or clinic* or outpatient* or out-patient* or non-medical or non-clinical or "patient care") SAME (personnel or staff or practitioner* or worker* or professional* or team* or officer* or assistant* or specialist* or workforce*))

11 32,670 #10 OR #9 OR #4 OR #3 OR #2 OR #1

10 229 TS=(UCINET or NetDraw or Pajek or KrackPlot)

9 723 #8 AND #7

8 196,284 TS=(network or networks)

7 1,955 #6 OR #5

6 1,123 TS="opinion leader*"

5 849 TS=(sociometr* or sociogram* or sociomap*)

4 22,133 TS=(("social network" or "social networks") SAME (intervention* or stud*))

3 25,273 TS=(("social network" or "social networks") SAME (method* or technique* or tool* or survey* or questionnaire* or diagram* or software or data or dataset* or measur* or metric* or indicator* or information))

2 21,583 TS=(("social network" or "social networks") SAME (theor* or framework* or model* or approach* or pattern* or perspective*))

1   25,557   TS=(("social network" or "social networks") SAME (analy* or investigat* or examin* or explor* or inquir*))

Key

TS= topic tag; searches terms in title, abstract, author keywords and keywords plus fields

* = truncation

$ = wildcard, unknown character or no character

“   “ = phrase search

SAME = terms within same sentence
